# Testing for alpha-1 antitrypsin in COPD in outpatient respiratory clinics in Spain: A multilevel, cross-sectional analysis of the EPOCONSUL study

**DOI:** 10.1371/journal.pone.0198777

**Published:** 2018-06-28

**Authors:** Myriam Calle Rubio, Joan B. Soriano, José Luis López- Campos, Juan J. Soler-Cataluña, Bernardino Alcázar Navarrete, José Miguel Rodríguez González- Moro, Marc Miravitlles, Miriam Barrecheguren, Manuel E. Fuentes Ferrer, Juan Luis Rodriguez Hermosa, on behalf of the EPOCONSUL Study

**Affiliations:** 1 Pulmonary Department, Instituto de Investigación Sanitaria del Hospital Clínico San Carlos (IdISSC), Hospital Clínico San Carlos, Madrid, España; 2 Departamento de Medicina, Facultad de Medicina, Universidad Complutense de Madrid, España; 3 Instituto de Investigación Hospital Universitario de la Princesa (IISP), Consultant on Methodology and Research of SEPAR, Universidad Autónoma de Madrid, Madrid, España; 4 Unidad Médico-Quirúrgica de Enfermedades Respiratorias. Instituto de Biomedicina de Sevilla (IBiS). Hospital Universitario Virgen del Rocío/Universidad de Sevilla, Sevilla, España; 5 CIBER de Enfermedades Respiratorias (CIBERES). Instituto de Salud Carlos III, Madrid. España; 6 Pulmonary Department, H. de Arnau de Vilanova, Valencia, España; 7 Pulmonary Department, Hospital de Alta Resolución de Loja, Granada, España; 8 Pulmonary Department, H. Universitario Principe de Asturias. Alcalá de Henares, Madrid, España; 9 Pulmonary Department, Hospital Universitari Vall d’Hebron, Barcelona, España; 10 CIBER de Enfermedades Respiratorias (CIBERES). Barcelona, España; 11 UGC de Medicina Preventiva, Hospital Clínico San Carlos, Instituto de Investigación Sanitaria del Hospital Clínico San Carlos (IdISSC), Madrid, España; National and Kapodistrian University of Athens, GREECE

## Abstract

**Background:**

Alpha-1 antitrypsin deficiency (AATD) is the most common hereditary disorder in adults, but is under-recognized. In Spain, the number of patients diagnosed with AATD is much lower than expected according to epidemiologic studies. The objectives of this study were to assess the frequency and determinants of testing serum α1-antitrypsin (AAT) levels in COPD patients, and to describe factors associated with testing.

**Methods:**

EPOCONSUL is a cross-sectional clinical audit, recruiting consecutive COPD cases over one year. The study evaluated serum AAT level determination in COPD patients and associations between individual, disease-related, and hospital characteristics.

**Results:**

A total of 4,405 clinical records for COPD patients from 57 Spanish hospitals were evaluated. Only 995 (22.5%) patients had serum AAT tested on some occasion. A number of patient characteristics (being male [OR 0.5, p < 0.001], ≤55 years old [OR 2.38, p<0.001], BMI≤21 kg/m2 [OR 1.71, p<0.001], FEV_1_(%)<50% [OR 1.35, p<0.001], chronic bronchitis [OR 0.79, p < 0.001], Charlson index ≥ 3 [OR 0.66, p < 0.001], or history or symptoms of asthma [OR 1.32, p<0.001]), and management at a specialized COPD outpatient clinic [OR 2.73,p<0.001] were identified as factors independently associated with ever testing COPD patients for AATD. Overall, 114 COPD patients (11.5% of those tested) had AATD. Of them, 26 (22.8%) patients had severe deficiency. Patients with AATD were younger, with a low pack-year index, and were more likely to have emphysema (p<0.05).

**Conclusion:**

Testing of AAT blood levels in COPD patients treated at outpatient respiratory clinics in Spain is infrequent. However, when tested, AATD (based on the serum AAT levels ≤100 mg/dL) is detected in one in five COPD patients. Efforts to optimize AATD case detection in COPD are needed.

## Introduction

Alpha-1antitrypsin deficiency (AATD) is the most common hereditary disorder in adults, characterized by impaired or defective production of the α1-antitrypsin (AAT) protein in the liver, which is associated with an increased risk of developing pulmonary emphysema and liver disease.

Recommendations of healthcare institutions such as the World Health Organization (WHO)[1], the Spanish National Guidelines for Chronic Obstructive Pulmonary Disease (GesEPOC)[2] and the American and European Thoracic/Respiratory Societies (ATS/ERS) [3,4] indicate that all chronic obstructive pulmonary disease (COPD)patients should be tested for AATD at least once during their lifetime. However, the real-life implementation of these clinical practice guidelines (CPG) is low [5]. AATD remains to be underdiagnosed despite the possibility available to determine AAT levels in blood and clinical guidelines recommendations [6,7].

The first step in the diagnosis of the deficiency is the determination of serum levels of AAT. In case of serum levels below the reference values, the next step will be the identification of the phenotype by immunoelectrophoresis or genotyping of the most frequent deficient alleles, according of the protocol of the local reference laboratory [8].

It is estimated that up to 2% of cases of COPD are associated with AATD. In particular, epidemiological studies in Spain estimate a total of 14,500 individuals with severe homozygous (PI*ZZ) deficiency [9], but only around 350 have been identified [10], suggesting that adherence to recommendations is poor. Potential benefits of case detection in all COPD patients include lifestyle recommendations (smoking prevention or cessation, avoiding high-risk occupations, limiting alcohol intake), identification of at-risk family members, genetic counselling and consideration for earlier augmentation therapy [4]. Understanding factors associated with better adherence to CPG recommendations to identify AATD in COPD is important in determining the possible reasons why such a delay in the diagnosis of AATD still exists.

The objective of our work was to investigate the frequency of determining AAT levels in COPD patients treated at outpatient respiratory clinics in Spain and to describe factors associated with AAT testing. We also aimed to study to determine the frequency of serum levels below the reference values of AAT in COPD patients treated at outpatient respiratory clinics in whom the test is performed and to describe clinical characteristics and data on referrals for diagnostic procedures carried out for COPD evaluation.

## Methods

The methodology of the EPOCONSUL audit has been extensively described elsewhere [11]. Briefly, the COPD audit promoted by the Spanish Society of Pulmonary and Thoracic Surgery (SEPAR) was designed to evaluate clinical practice as well as clinical and organizational factors related to managing patients with COPD across Spain. It was designed as an observational cross-sectional study. Recruitment was intermittent over a year (May 2014–May 2015). Every 2 months, each investigator recruited the clinical records of the first 10 patients identified as being diagnosed with COPD and seen in the outpatient respiratory clinic. Subsequently, the patients identified were reevaluated to determine if they met the inclusion/exclusion criteria described in S1 Table. Of the 175 public hospitals in the National Health System invited by SEPAR, 59 participated (33.3%). The estimated reference population for the EPOCONSUL study was 18,104,350 inhabitants, representing 39% of the Spanish population. The distribution of hospitals in the different regions and participating investigators are included in S1 Appendix.

The information collected was historical in nature for the clinical data of the last and previous visits, and information about hospital resources was concurrent. Data from this study in S1 File.

In order to compare hospitals, they were divided into two types of center according to their size (small or large) as measured by: the number of beds per center ≥500, the number of inpatient respiratory beds ≥20, the number of pulmonology staff members ≥5, and the number of annual outpatient respiratory visits ≥10,000. All the criteria must be met to be considered large.

In order to evaluate the degree of current CPG implementation of the recommendation (determining AAT blood levels in all COPD patients), the cases audited from 57 hospitals with available AAT blood level determination were analyzed.

Based on the serum AAT levels, individuals were classified as follows: no deficiency: AAT >100 mg/dL; intermediate deficiency: AAT between 51 mg/dL and 100 mg/dL; and severe deficiency: AAT ≤50 mg/dL [12].

The patients with clinical/radiological or functional characteristics of emphysema were classified as emphysema phenotype.

The protocol was approved by the Ethics Committee of the Hospital Clínico San Carlos (Madrid, Spain; internal code 14/030-E). Additionally, according to current research laws in Spain, the ethics committee at each participating hospital evaluated and agreed to the study protocol. The need for informed consent was waived due to the non-interventional nature of the study, the anonymization of data and the need to blindly evaluate the clinical performance. This circumstance was clearly explained in the protocol, and the ethical committees approved this procedure. To avoid modifications to the usual clinical practice and preserve the blinding of the clinical performance evaluation, the medical staff responsible for the outpatient respiratory clinic was not informed about the audit. Data was entered remotely at each participating location to a centrally-controlled server.

## Statistical analysis

Qualitative variables were summarized by their frequency distribution and quantitative variables by their median, interquartile range (IQR) and minimum–maximum. The differences between hospital resources and characteristics according to size (small vs large) were evaluated using χ2 tests for categorical data, while the non-parametric Mann-Whitney test was used for continuous data. With regard to missing data, after performing data cleansing to identify and correct missing and extremely unlikely values, the data was included in the analysis as missing information.

The association between each independent variable (patient characteristics, hospital resources and work organization) and the dependent variable AAT level determination was assessed by calculating the crude odds ratio (OR) via mixed effect logistic regression model. Each model included a random effect to account for the correlation between individuals within the same hospital. A multivariable mixed effect model was fitted in order to evaluate the independent effect of the selected variables. Candidate predictors with a value of *p* < 0.10 in the univariate analysis were accepted for inclusion in the multilevel multivariate analysis. Variables were removed from the model when the *p*-value exceeded 0.10 and were kept in the final model when less than 0.05.

All analyses were performed using STATA 12.0 software. Statistical significance was assumed as *p* < 0.05.

## Results

### Population

**Fig 1:**
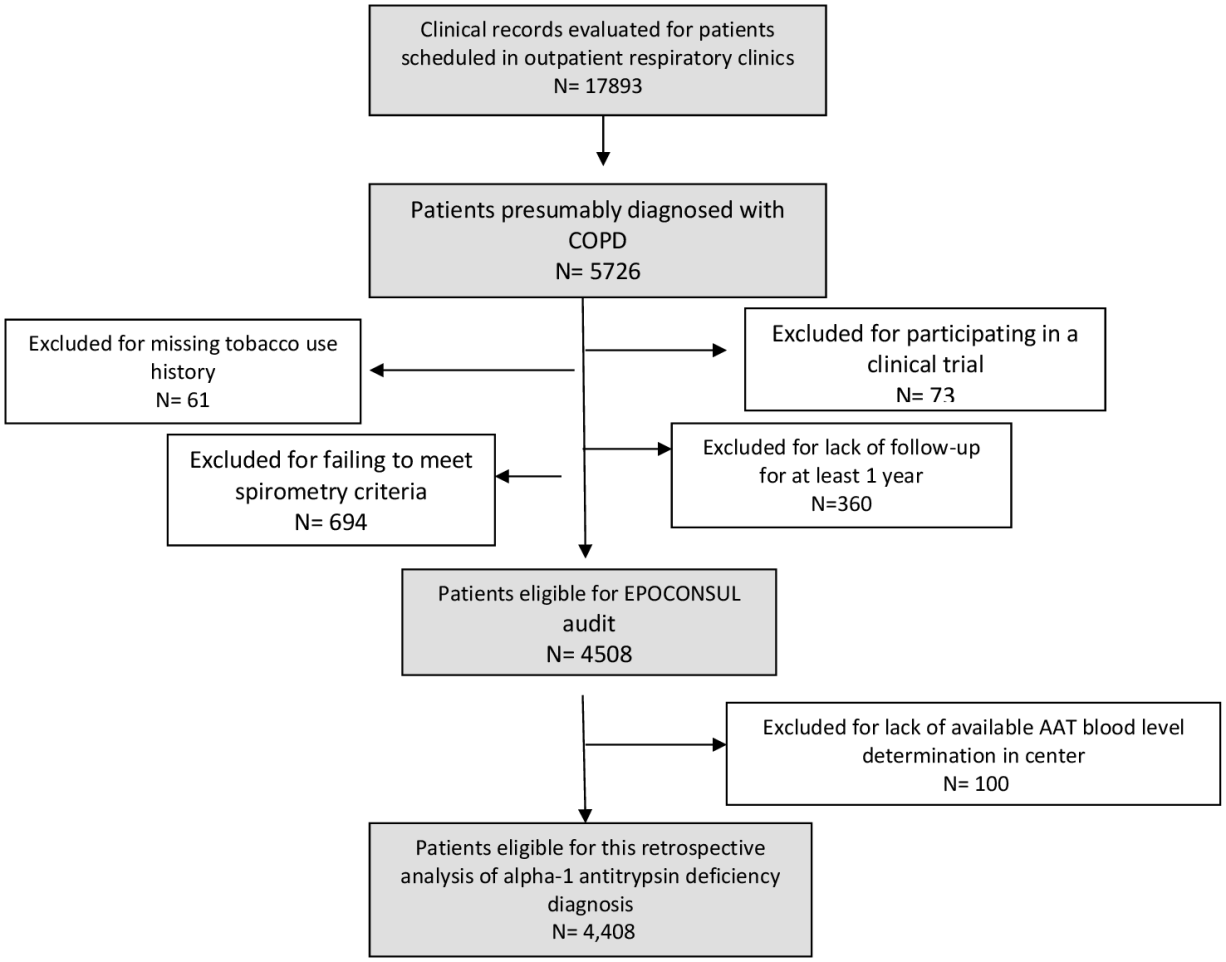
The sampling process is described in a STROBE flow chart. A total of 17,893 clinical records of patients treated in outpatient respiratory clinics were evaluated during the study period and 5,726 clinical records of patients presumably diagnosed with COPD were selected. Of them, 4,508 patients were audited from 59 hospitals, for having all the inclusion criteria and none of the exclusion criteria. A total of 4408 patients audited from 57 hospitals with available AAT blood level determination were included for this retrospective analysis of alpha-1 antitrypsin deficiency diagnosis.

**Fig 2:**
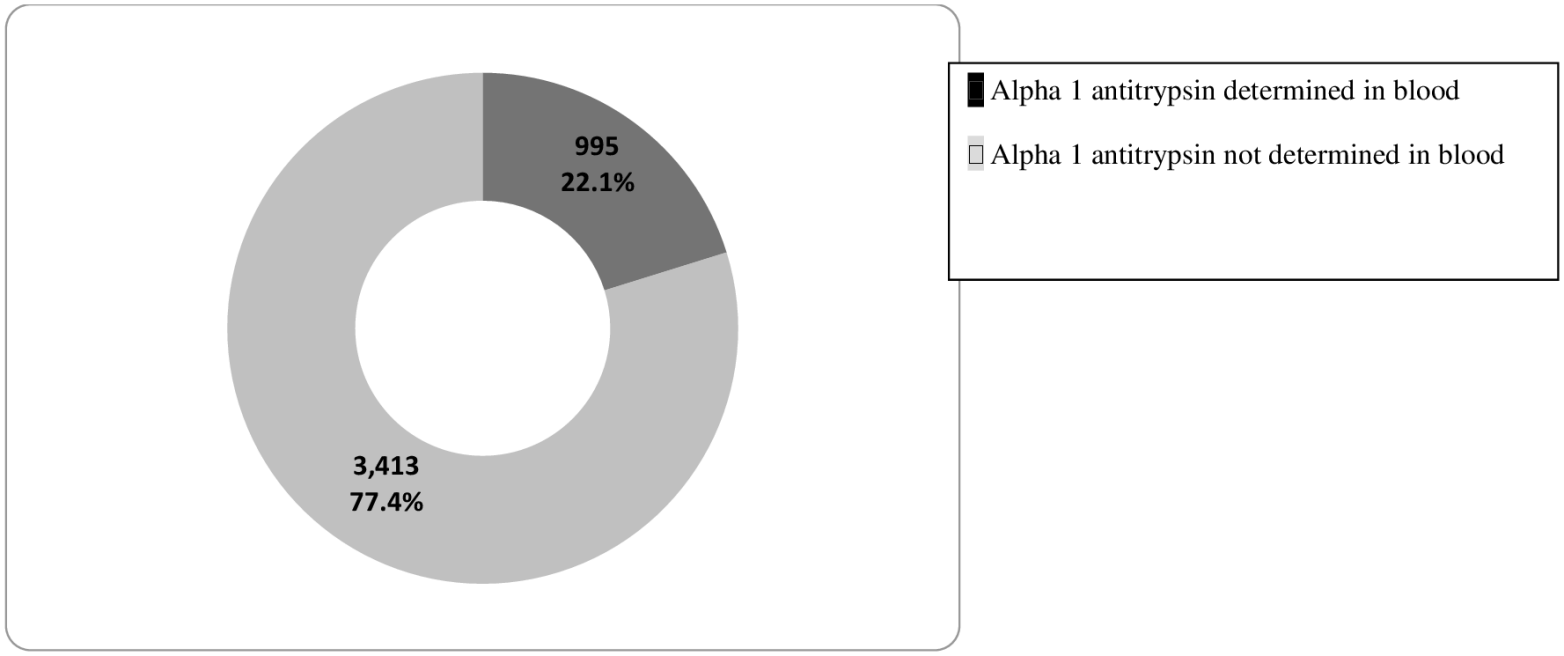
Distribution of AAT level determination in the blood. Of the analyzed cohort, only 995 (22.5%) patients underwent AAT determination.

A total of 4408 patients audited from 57 hospitals were included in the analysis. The sampling process was detailed in an epidemiology flow chart and described in Fig 1. Of the analyzed cohort, only 995 (22.5%) patients underwent AAT determination (Fig 2).

### Characteristics of the individuals tested for AAT concentrations

The mean AAT plasma level was 143 (SD 41.6) mg/dL. In total, 114 patients (11.5% of the individuals tested) had the serum AAT levels ≤100 mg/dL. Of them, 88 (77.2%) patients had the serum AAT levels between 51 mg/dL and 100 mg/dL (might be considered an intermediate AATD), while 26 (22.8%) patients had the serum AAT levels≤ 50mg/dl (a severe AATD).

Patients with AATD (based on the serum AAT levels ≤100 mg/dL) were younger than individuals with normal AAT levels (61.7 years vs 66 years, p<0.001), with a lower pack-year index (39 versus 45, p<0.001), were more likely to have characteristics of emphysema (34.3% versus 18.9%, p = 0.003), have less dyspnea and were less likely to have a Charlson index≥3 (23.7% vs 39.7%,p<0.001). During follow-up in patients with AATD, diffusing capacity (78.9% vs 68.7%, p = 0.024) and lung volume (74.6% vs 62.7%, p = 0.013) were more commonly measured and the 6-minute walk test more frequently carried out (48.2% vs 38.1%, p = 0.038), as summarized in Table 1.

**Table 1:** Characteristics of COPD patients tested for AAT, and diagnostic procedures conducted during the follow-up for medical evaluation according to presence/absence of AATD.

**Characteristics of the patient**	**No AATD** **(AAT >100mg/dl)** **(n = 881)**	**AATD** **(AAT ≤ 100mg/dl)** **(n = 114)**	**P**
**Serum AAT level (mg/dl), m (SD)**	149.95 (34.9)	56.18 (25)	
**Age (years), m (SD)**	66.05 (9.6)	61.73 (10.2)	<0.001
**Sex (female), (%)**	21.1	19.3	0.654
**Current smoker, (%)**	25.4	21.9	0.417
**Smoking pack-year, median (IQR)**	45 (33–70)	39 (25–55)	<0.001
**BMI kg/m2, m (SD)**	27.12 (5.3)	26.70 (5.2)	0.435
**Dyspnea (mMRC)**			0.050
**0–1 (%)**	32.7	20.2	
**≥2 (%)**	42.5	51.8	
**Charlson index ≥3, (%)**	39.7	23.7	0.001
**Chronic bronchitis, (%)**	38.6	33.3	0.276
**Emphysema phenotype, (%)**	18.9	34.3	0.003
**History of asthma or symptoms suggestive of asthma, (%)**	34.1	28.9	0.277
**COPD Phenotype**			0.146
**Non-exacerbator, (%)**	36.2	32.5	
**Exacerbator, (%)**	20.9	28.9	
**Number of hospitalizations in the last year, (%)**			0.615
**0**	77.8	79.8	
**≥1**	22.2	20.2	
**%predicted post-BD FEV**_**1**_**, m (SD)**	48.56 (18.3)	48.38 (18.9)	0.921
**<50%, (%)**	55.7	50.9	
**BODE, m (SD)**	4.26 (2.0)	4.66 (1.6)	0.303
**Long-term oxygen therapy, (%)**	26.3	38.6	0.006
**Diagnostic procedures conducted during the follow-up for COPD evaluation**	**No AATD** **(AAT >100mg/dl)** **(n = 881)**	**AATD** **(AAT ≤ 100mg/dl)** **(n = 114)**	**P**
**Diffusing capacity measured, (%)**	68.7	78.9	0.024
**Lung volumes measured, (%)**	62.7	74.6	0.013
**6-minute walk test performed, (%)**	38.1	48.2	0.038
**BODE index registered, (%)**	20.4	28.1	0.061
**Chest CT scan done, (%)**	73.4	80.7	0.095

Non-exacerbator: patients with 0 or 1 exacerbation in the previous year; Exacerbator: patients who experienced at least two exacerbations in the previous year.

### Center characteristics

The hospital characteristics and respiratory unit resources are summarized in S2 Table. Large hospitals constituted 56% of centers. The majority of participating centers were public (93%), university hospitals (86%) and had a pulmonology resident available (70.2%). Although the larger hospitals had more staff (median 13; CI 95% 10–16 vs median 6; CI 95% 2–8, p <0.001), the availability of respiratory unit resources was similar, including AATD genotyping (71.9% vs 68%, p = 0.751). There were few centers with a specialized COPD outpatient clinic (47.4%), regardless of hospital size.

### Center and respiratory unit resource characteristic sand their association with AAT testing in COPD patients

The majority of center-level variables were not associated with adherence to the recommendation to test for AAT in patients with a diagnosis of COPD, except the number of annual outpatient respiratory visits ≥10,000 (OR 2.18, p = 0.05) and the availability of a specialized COPD outpatient clinic (OR 1.91, p = 0.021). Table 2 describes the logistic regression univariate analysis with the variables related to centers.

**Table 2:** Characteristics of participating hospitals and respiratory unit resources, by COPD patients with/without serum AAT levels tested.

Centers	All patients (n = 4,408)	COPD patients with serum AAT levels tested (n = 995)	COPD patients without serum AAT levels tested (n = 3,413)	OR (95%CI)	p
Large hospital, (%)	62.9	67.4	61.6	1.41 (0.79–2.50)	0.240
University hospital, (%)	88	88.5	87.9	1.02 (0.44–2.32)	0.962
Beds per center ≥500, (%)	71.9	74.2	71.2	1.18 (0.64–2.17)	0.581
Respiratory ward not available, (%)	11.5	8.1	12.5	Reference	
Respiratory ward<20 beds	13.4	12.9	13.5	1.46 (0.49–4.31)	0.485
Respiratory ward≥20 beds	75	78.9	73.8	1.58 (0.68–3.67)	0.278
Number of pulmonology staff members ≥5, (%)	78.9	80.2	78.5	0.80 (0.35–1.86)	0.620
Pulmonology residents present, (%)	74.1	75.2	73.8	1.01 (0.53–1.91)	0.963
Number of annual outpatient respiratory visits ≥ 10,000, (%)	88.8	93.3	87.4	2.18 (0.99–4.79)	0.05
≥ 15 minutes of follow-up at general outpatient respiratory visit, (%)	41.9	42.9	417	1.16 (0.65–2.06)	0.618
AATD genotyping availability, (%)	74.1	77.3	73.2	1.41 (0.75–2.65)	0.284
Specialized COPD outpatient clinic available, (%)	52.5	61.4	50	1.91 (1.10–3.32)	0.021
Outpatient respiratory nursing clinic availability, (%)	45.9	50.4	44.5	1.32 (0.75–2.33)	0.333
Inhalation technique educational program available, (%)	29.9	33.8	28.8	1.23 (0.67–2.28)	0.494

The necessary criteria to consider a hospital large are: the number of beds per center ≥500, the number of inpatient respiratory beds ≥20, the number of pulmonology staff members ≥5, and the number of annual outpatient respiratory visits ≥10,000.

### Sociodemographic and clinical patient characteristics and their association with AAT testing in COPD patients

Table 3 describes the characteristics of the evaluated patients and the association with AAT testing in COPD patients (logistic regression univariate analysis). A large number of the patient-level variables were associated with AAT level determination, such as being male (OR 0.47, p<0.001), age ≤55 years old (OR 3.16, p<0.001), pack-year index (10 pack-years) (OR 0.97, p<0.043),being an active smoker (OR 1.30, p = 0.004), BMI≤21 (OR 2.21, p<0.001), Charlson index ≥3 (OR 0.57, p<0.001), symptoms of asthma (OR 1.55, p<0.001), and a FEV1 <50% (OR 1.33, p<0.001). Being treated at a specialized COPD outpatient clinic was associated with a higher likelihood of being tested for AAT (OR 2.94, p<0.001).

**Table 3:** Characteristics of COPD patients and hospital care variables according to whether serum AAT levels were determined to detect cases of AATD (logistic regression bivariate analysis).

	All patients (n = 4.408)	COPD patients with serum AAT levels tested (n = 995)	COPD patients without serum AAT levels tested (n = 3,413)	OR (95%CI)	p
**Sex (male), (%)**	85.7	79	87.6	0.47 (0.38-.58)	<0.001
**Age (years), m (SD)**	69.66 (9.77)	65.55 (9.82)	70.86 (9.42)	0.94 (0.93–0.95)	<0.001
**Patients ≤55 (%)**	8.6	15.87	6.50	3.16 (2.47–4.04)	<0.001
**Smoking pack-year, m (SD)**	55.9 (33.74)	54.10 (32.06)	56.46 (34.19)	0.97 (0.95–0.99)	0.043
**Active smokers, (%)**	23.3	25	22.8	1.30 (1.08–1.55)	0.004
**BMI kg/m2, m (SD)**	28.03 (5.45)	27.07 (5.37)	28.31 (5.45)	0.94 (0.92–0.95)	<0.001
**≤21 (%)**	7.1	11.1	6.9	2.21 (1.68–2.91)	<0.001
**Charlson index≥3, (%)**	45.2	37.8	47.4	0.57 (0.49–0.67)	<0.001
**Dyspnea (mMRC)**					
**0–1, (%)**	27	31.2	25.7	Reference	
**≥2, (%)**	41.1	43.5	40.4	0.88 (0.73–1.07)	0.797
**Missing, (%)**	13.4	10.6	14.2	0.43 (0.32–0.58)	<0.001
**Level of dyspnea not referred to, (%)**	18.3	14.5	19.5	0.50 (0.38–0.66)	<0.001
**Chronic bronchitis, (%)**	41.4	38	42.4	0.85 (0.72–1.00)	0.056
**History of asthma, or symptoms suggestive of asthma,(%)**	26.5	33.5	24.5	1.55 (1.29–1.86)	<0.001
**Emphysema phenotype, (%)**	18.7	20.8	17.8	1.27 (0.97–1.66)	0.079
**%predicted post-BD FEV**_**1**_**, m (SD)**	50.96 (17.7)	48.54(18.3)	51.66 (17.4)	0.99 (0.98–1)	<0.001
**<50%**	49	55.1	47.3	1.33 (1.14–1.56)	<0.001
**COPD Phenotype**					
**Non-exacerbator, (%)**	26.7	35.7	24	Reference	
**Exacerbator, (%)**	18.6	21.8	17.7	0.84 (0.67–1.04)	0.119
**Missing, (%)**	54.6	42.4	58.1	0.45 (0.37–0.55)	<0.001
**Number of hospital admissions in the last year≥1, (%)**	23.3	22	23.7	0.91 (0.75–1.10)	0.367
**Chronic colonization, (%)**	6	5.9	6	1.15 (0.83–1.60)	0.381
**Triple or quadruple inhaled therapy, (%)**	66.9	61	65.7	1.18 (1.00–1.39)	0.039
**Long-term oxygen therapy, (%)**	26.6	27.7	26.2	1.05 (0.88–1.26)	0.544
**Home ventilation, (%)**	7.5	6.8	7.9	0.69 (0.50–0.96)	0.027
**Type of respiratory outpatient clinic:**					<0.001
**general clinic, (%)** **specialized COPD clinic, (%)**	47.4	38.6	49.9	1	
	52.6	61.4	50.1	2.94 (2.26–3.83)	
**Respiratory care follow-up (years), median (IQR)**	4(2–7)	4 (2–6)	4 (2–7)	1.00 (0.99–1.00)	0.382

Non-exacerbator: patients with 0 or 1 exacerbation in the previous year; Exacerbator: patients who experienced at least two exacerbations in the previous year; Dyspnea not quantified: level of dyspnea not referred to. Abbreviations: BMI: body mass index; mMRC, modified Medical Research Council; %predicted post-BD FEV_1_, percent predicted post-bronchodilator FEV_1_; GesEPOC: Spanish National Guidelines for COPD. IQR: interquartilerange.

### Multilevel analysis of factors associated with AAT testing in COPD patients

In the adjusted model, summarized in Table 4, being ≤55 years old (OR 2.38, p<0.001), having a BMI≤21 (OR 1.71, p<0.001), FEV1<50% (OR 1.75, p<0.001), history or symptoms of asthma (OR 1.32, p<0.001) and being treated at a specialized COPD outpatient clinic (OR 2.73, p<0.001) were positively associated with AAT testing, while Charlson index≥3 (OR 0.66, p<0.001), being male (OR 0.55, p<0.001) and chronic bronchitis criteria (OR 0.79, p<0.001) showed a statistically significant negative association. Some unrecorded values (COPD phenotype missing, dyspnea missing, or level of dyspnea not referred to) showed a significant negative association.

**Table 4:** Multivariate logistic regression to identify independent factors associated with testing of COPD patients for serum AAT levels to detect cases of AATD.

Variable	OR (95%CI)	p
Sex (male)	0.55 (0.44–0.69)	<0.001
Age ≤55(years)	2.38 (1.82–3.13)	<0.001
Charlson index≥3	0.66 (0.55–0.78)	<0.001
Chronic bronchitis	0.79 (0.66–0.95)	<0.001
Medical history of asthma, or symptoms suggestive of asthma	1.32 (1.08–1.61)	<0.001
Dyspnea (mMRC)		
0–1, (%)	1	
≥2, (%)	0.86 (0.68–1.07)	0.185
Missing, (%)	0.47 (0.29–0.64)	<0.001
Level of dyspnea not referred to,(%)	0.53 (0.37–0.71)	<0.001
BMI ≤21 kg/m2	1.71 (1.27–2.29)	<0.001
% predicted FEV_1_<50%	1.35 (1.13–1.62)	<0.001
COPD phenotype		
Non-exacerbator, (%)	1	
Exacerbator, (%)	0.91 ((0.55–1.17)	0.495
Missing, (%)	0.59 (0.35–0.73)	<0.001
Managed at a specialized COPD outpatient clinic	2.73 (2.07–3.61)	<0.001
Triple or quadruple inhaled therapy, (%)	1.17 (0.90–0.98)	0.09
Home ventilation, (%)	0.70 (0.60–0.98)	0.04

## Discussion

Our results have shown that only 22.5% of COPD patients followed up in hospitals in Spain have a AAT value recorded in their clinical records. This study provides data for the first time about the frequency with which AAT is tested in COPD patients treated in outpatient respiratory clinics in Spain, as well as factors associated with performing this test.

One of most relevant aspects with regard to AATD is that, despite the fact it is considered the most common hereditary disease in adults and is potentially fatal, the disease continues to be underdiagnosed around the world [12,13].

In Spain, the S and Z allele frequency are estimated to be 104/1.000 and 17/1.000 respectively, which, taking the Spanish population into account, means that 14.500 people have a ZZ phenotype and 175.000 have an SZ phenotype [14,15]. These data places Spain as the country with the second highest number of severe AATD in Europe after Italy [14]. However, the prevalence of AATD clashes with the reality of the Spanish Registry of Patients with Alpha-1 Antitrypsin Deficit (REDAAT) that only registered 348 individuals Pi*ZZ and 100 Pi*SZ, representing less than 1% of existing cases [10,16]. Additionally, studies have estimated a 5–10 year delay between the diagnoses of COPD and AATD in Spain [6,13].

The underdiagnosis of AATD is a current reality, which is supported by the results of this study, making it clear that the majority of COPD patients treated in outpatient respiratory clinics in Spain did not undergo AAT determination in blood in spite of having prolonged follow-up. These data reflects the inadequate clinical practice with regard to detecting cases of AATD in an at-risk group such as COPD patients and does not adhere to CPG recommendations, which calls for determining AAT levels in blood for all patients with a COPD diagnosis [1–4].

In our study, we found a rate of only 22.6 determinations of AAT levels in blood per 100 COPD patients treated in an outpatient respiratory clinic in centers where this test was available, signaling a lack of awareness and understanding of AATD among many doctors, who forget to request serum AAT levels in the majority of their COPD patients as the main determining factor in this underdiagnosis. This poor awareness about AATD is also present in primary care. A recent population-based study with data from electronic clinical records from primary care in Catalonia revealed a frequency of AAT testing of only 4–6 determinations per 10,000 inhabitants, both in children and adults between the years 2007 and 2011. This study also showed that patients with detected AATD were not always referred to a specialist [17].

Studies on the level of training for AATD have shown poor knowledge among doctors, even those interested in respiratory diseases [18], identifying the erroneous perception of the high cost of the test or the need to refer the patients to other specialists in order to make the diagnosis as the main reasons for not determining AAT levels in blood [19]. In a survey done in Spain and Portugal, only 14% of doctors surveyed referred to having “a good level” of knowledge on AATD and only 55% of pulmonologists knew the AAT threshold considered to be a severe deficiency, which would indicate that many doctors’ knowledge about how to make the diagnosis is insufficient. More importantly, only 27% of respiratory specialists declared to systematically test all their COPD patients for AAT [20].

With regard to the factors associated with determining testing for AAT found in our study, it is striking that the majority were patient-level variables. These associated factors would be consistent with the typical clinical characteristics classically attributed to AATD-related COPD and could be summarized as “a young adult with progressive dyspnea and developed emphysema despite lower tobacco exposure”. This could reflect suspected AATD in a patient with a “typical” profile, thus a focused case-detection is done. However, with only these criteria, multiple other types of patients with less typical presentation would not be diagnosed. For this reason, since 1997, the WHO [1] consensus document has recommended that AAT concentrations be determined in all COPD patients at least once in their lives, a recommendation maintained in GesEPOC [2] and international CPG (ATS/ERS) [3,4].

It is also important to consider that being treated in a clinic specialized in COPD was associated with a higher likelihood of determining AAT levels in blood in our study. This was an interesting result that could be related to a higher awareness or interest among professionals treating patients in specialized clinics, or due to the use of electronic medical registries.

Although patients in whom AAT was determined came from centers with a higher volume of patients and greater availability of clinics specialized in COPD, none of the characteristics of the center or pulmonology unit resources were maintained as independent factors associated with testing for AATD in the multivariate analysis.

With regard to the diagnosis of AATD, in Spanish screening or case-detection programs in COPD patients, such as that done in 2005 by de la Roza et al. where, out of 971 samples, 62 (6.4%) had low levels of AAT and only 4 patients had a severe deficiency [21], or the IDDEA project (Information and Detection of Alpha-1-Antitrypsin Deficit) carried out in 2008 by the REDAAT in Primary Care, where of the 596 samples from COPD patients, AAT levels < 60 mg/dl were detected in only 6 (1.1%) [22]. However, in another study conducted in Italy, of a total of 1841 patients, 151 (8.2%) were identified as having a severe deficiency [23]. This disparity in AATD detection rates is likely due to the inclusion criteria of the study populations. In our analysis, though we find 2.61 cases of severe AATD (based on the serum AAT levels ≤50 mg/dL) for every 100 AAT determinations, that can consider higher, but it is necessary to remember that individuals tested for AAT concentrations was not systematic and is affected by the choice of the clinicians. Patients identified with AATD, based on the serum AAT levels ≤100 mg/dL, were younger, with less tobacco use and clinical characteristics of emphysema. However, there were no differences in airflow limitation severity or BODE index, which suggests a late diagnosis of AATD.

It is of note that more specific respiratory function diagnostic tests, such as lung volume measurement, diffusing tests and the 6-minute walking test were more commonly requested in patients with AATD.

Our study has some strengths and limitation. The main strength is the sample size, which accounts for 39% of the Spanish population. Nevertheless, the limitations to be considered are the fact that the centers were not randomly selected and were chosen based on having participated previously in COPD clinical audits and their interest in the study. Additionally, as in all clinical audits, despite inclusion methodology and periodic supervision of the database, some of the values were not included as they were not available. We must also mention the possible limitation in the diagnosis of the deficiency of AAT only based on concentration of the protein in blood, that might account for temporary deficiency due to liver diseases, low-protein diet, hormonal unbalance. Too we must keep in mind that, as an acute phase reactant, infectious or inflammatory processes may alter AAT determination [24], thus normal or high values may be detected in individuals with an intermediate deficiency. However, despite these limitations, the sample included is likely representative of medical attention for patients with COPD in outpatient respiratory clinics in Spain.

## Conclusions

Testing for AAT in COPD patients treated in outpatient respiratory clinics is done infrequently. The presence of clinical characteristics typical of AATD-related COPD is a factor associated with testing, which suggests that a selective search strategy is used in clinical practice, despite what is recommended in current guidelines. Additionally, being treated in a clinic specialized in COPD is a factor associated with greater frequency in determining AAT, which could be explained by the greater interest and knowledge among professionals.

Determining AAT in all patients with COPD is the key to improving underdiagnosis and achieving early-stage diagnosis. As a result, it is necessary to establish training and awareness programs for healthcare professionals and to use electronic medical records in usual clinical practice as support tools to avoid variability and improve quality of care. Efforts to optimize AATD case detection in COPD are needed.

## Supporting information

S1 Table The inclusion criteria and exclusion criteria. (DOC)

S2 Table Characteristics of the participating hospitals and resources of the respiratory units. (DOC)

S1 Appendix Participants investigators in EPOCONSUL study. (DOC)

S1 File Data from this study. (XLS)
