# Correction: Testing for alpha-1 antitrypsin in COPD in outpatient respiratory clinics in Spain: A multilevel, cross-sectional analysis of the EPOCONSUL study

**DOI:** 10.1371/journal.pone.0212522

**Published:** 2019-02-12

**Authors:** Myriam Calle Rubio, Joan B. Soriano, José Luis López- Campos, Juan J. Soler-Cataluña, Bernardino Alcázar Navarrete, José Miguel Rodríguez González- Moro, Marc Miravitlles, Miriam Barrecheguren, Manuel E. Fuentes Ferrer, Juan Luis Rodriguez Hermosa, on behalf of the EPOCONSUL Study

The following information is missing from the Competing Interests section:

JBS declares having received grants from 2014 to date from Linde via Hospital Universitario de La Princesa and has participated in speaking activities, advisory committees and consultancies during the period 2014−2018 sponsored by Almirall, AstraZeneca, Boehringer Ingelheim, CHEST, Chiesi, ERS, Esteve, GEBRO, Grifols, GSK, Linde, Lipopharma, Mundipharma, Novartis, Pfizer, RiRL, Rovi, Sandoz, SEPAR and Takeda, outside the submitted work. JLLC has received fees for giving conferences, scientific advising, participation in clinical studies or draft of publications for (alphabetical order): Almirall, AstraZeneca, Bayer, Boehringer Ingelheim, Cantabria Pharma, Chiesi, Esteve, Faes, Ferrer, Gebro, GlaxoSmithKline, Grifols, Menarini, MSD, Novartis, Pfizer, Rovi, Teva y Takeda. JJSC has received speaker fees from Almirall, AstraZeneca, Boehringer Ingelheim, Chiesi, Esteve, Ferrer, GSK, Menarini, Mundipharma, Novartis, Rovi, consulting fees from Almirall, AstraZeneca, Boehringer Ingelheim, Chiesi, Esteve, Gebro, GSK, Mundipharma and Novartis and grants from Boehringer Ingelheim, GSK, Chiesi and Novartis. BAN reports personal fees from GSK, Gebro, and Astra- Zeneca; grants, personal fees and non-financial support from Novartis AG and Laboratorios Menarini; and personal fees and non-financial support from Boehringer Ingelheim and Chiesi outside the submitted work. JMRGM has received speaker fees from AstraZeneca, Boehringer Ingelheim, Chiesi, Esteve, GSK, Menarini, and Novartis and consulting fees from Boehringer Ingelheim and GSK. MM has received speaker or consulting fees from AstraZeneca, Bial, Boehringer Ingelheim, Chiesi, Cipla, CSL Behring, Laboratorios Esteve, Gebro Pharma, GlaxoSmithKline, Grifols, Menarini, Mereo Biopharma, Novartis, pH Pharma, Rovi, TEVA, Verona Pharma and Zambon, and research grants from GlaxoSmithKline and Grifols. MB has received speaker fees from Grifols, Menarini, CSL Behring, and GSK and consulting fees from Novartis, GSK and Gebro Pharma. AstraZeneca has several products in development or marketed products related to COPD.
