# Supplementary material for: Testing for alpha-1 antitrypsin in COPD in outpatient respiratory clinics in Spain: A multilevel, cross-sectional analysis of the EPOCONSUL study
Source: PLoS One. 2018 Jun 28;13(6):e0198777. doi: 10.1371/journal.pone.0198777 (PMC6023216; doi:10.1371/journal.pone.0198777)
Supplement: S1 Table — (DOC) [file pone.0198777.s001.doc]

**S1 Table:**  **The inclusion criteria and exclusion criteria**

| The inclusion criteria | - patients aged ≥40 years  - smokers or ex-smokers (of at least 10 pack-years)  - COPD diagnosed on the basis of spiro­metric tests (FEV1/FVC post-bronchodilation<0.7 or FEV1/FVC pre-bronchodilation<0.7 and FEV1≥80%, if there is no bronchodilation reversibility testing available |
| --- | --- |
| The exclusion criteria | - lack of follow-up for at least 1 year in a respiratory outpatient clinic  - participating in a clinical trial |
