# Supplementary material for: Testing for alpha-1 antitrypsin in COPD in outpatient respiratory clinics in Spain: A multilevel, cross-sectional analysis of the EPOCONSUL study
Source: PLoS One. 2018 Jun 28;13(6):e0198777. doi: 10.1371/journal.pone.0198777 (PMC6023216; doi:10.1371/journal.pone.0198777)
Supplement: S2 Table — (DOC) [file pone.0198777.s002.doc]

**S2 Table: Characteristics of the participating hospitals and resources of the respiratory units**

|  | **All** | **Small hospital** | **Large hospital** | **P**† |
| --- | --- | --- | --- | --- |
| **Number of participating hospitals, n** | **57** | **25** | **32** |  |
| **Public hospital (%)** | 93 | 84 | 100 | 0.032 |
| **University hospital (%)** | 86 | 68 | 100 | 0.001 |
| **Beds per center, median (P25-75)** | 651 (349-943) | 349 (255-450) | 903 (702-1199) | <0.001 |
| **Number of pulmonology staff members, median (P25-75)** | 10 (5-13) | 6 (2-8) | 13 (10-16) | <0.001 |
| **Pulmonology residents available (%)** | 70.2 | 36 | 96.9 | <0.001 |
| **Number of annual outpatient respiratory visits, median (IQR)** | 15690  (12004-25680) | 12345  (4402-13606) | 23985  (16070-27838) | <0.001 |
| **≥ 15 minutes of follow-up at general outpatient respiratory visit (%)** | 43.9 | 48 | 40.6 | 0.578 |
| **Specialized COPD outpatient clinic available (%)** | 47.4 | 40 | 53.1 | 0.425 |
| **Outpatient respiratory nursing clinic availability (%)** | 47.4 | 48 | 46.9 | 1.000 |
| **Functional respiratory laboratory available (%)**   - **Spirometry** - **Diffusing capacity** - **Plethysmography** - **Respiratory muscle strength** - **6MWT available** - **Cardiopulmonary exercise testing available** | 100  100  100  84.2  96.5  63.2 | 100  100  100  64  92  40 | 100  100  100  100  100  81.3 | 1  1  1  <0.001  0.188  0.002 |
| **Genotyping of AATD availability** | 70.2 | 68 | 71.9 | 0.751 |
| **Inhalation technique educational program available (%)** | 14 | 20 | 9.4 | 0.280 |
| **Respiratory rehabilitation program available (%)**   - **Hospital-based** - **Home-based** - **Mixed** | 75.4  60.5  7  32.6 | 68  58.8  11.8  29.4 | 81.3  61.5  3.8  34.6 | 0.249  0.607 |

**Notes:**

The criteria that were necessary to be considered center large were the number of beds per center ≥500, the number of inpatient respiratory beds ≥20, the number of pulmonology staff members ≥5 and the number of annual outpatient respiratory visits ≥10,000. All the criteria are necessary to be considered large.

**Abreviature**s: AATD: alpha-1 antitrypsin deficiency; 6MWT: 6-minute walk test; IQR: interquartile range.
