# Supplementary material for: Testing for alpha-1 antitrypsin in COPD in outpatient respiratory clinics in Spain: A multilevel, cross-sectional analysis of the EPOCONSUL study
Source: PLoS One. 2018 Jun 28;13(6):e0198777. doi: 10.1371/journal.pone.0198777 (PMC6023216; doi:10.1371/journal.pone.0198777)
Supplement: S1 Appendix — (DOC) [file pone.0198777.s003.doc]

# Supporting Information

**S1 Appendix: Participants investigators in EPOCONSUL study**

*Andalucía*: Jose Luis Rojas Box, H. de Alta Resolución de Écija, Sevilla. Jose Domingo Garcia Jimenez, H. de Alta Resolución de Utrera, Sevilla. Adolfo Domenech del Rio, Ana Muñoz. H. Carlos Hayas, Málaga. Antonia Soto Venegas, H. San Juan de la Cruz, Úbeda, Jaén. Aurelio Arnedillo Muñoz. H. U. Puerta del Mar, Cádiz. Agustín Valido Morales. H. Virgen de Macarena. Sevilla. Jose Velasco Garrido, Carlos Rueda Ríos, Macarena Arroyo Varela H. Virgen de la Victoria. Málaga. Francisco Ortega Ruiz, Eduardo Marquez Martin, Carmen Calero Acuña, H. Virgen del Rocio, Sevilla. Francisco Luis Garcia Gil, H. U Reina Sofia, Córdoba.

*Aragón*: Joaquin Carlos Costan Galicia, H. Clínico U. Lozano Blesa, Zaragoza. Ana Boldova Loscertales, H. Royo Villanova, Zaragoza.

*Asturias*: Cristina Martinez González, Rosirys Guzman Taveras, H. U. Central de Asturias, Oviedo.

*Murcia*: Juan Luis De la Torre Alvaro, H. U Santa Lucia, Cartagena, Mª Jesus Avilés Ingles, H. General U. Reina Sofia, Murcia. Rubén Andújar Espinosa, H.U. Virgen de la Arrixaca, Murcia.

*Canarias*: Juan Manuel Palmero Tejera, Juan Marco Figueira Conçalves, H.U. Nuestra Señora de la Candelaria, Santa Cruz de Tenerife.

*Cantabria*: Ramon Agüero Balbín, Carlos Amado Diago, Beatriz Abascal Bolado.

H. Marqués de Valdecilla, Santander. Juan Luis Garcia Rivero, Marcelle Cohen Escovar, H. de Laredo, Santander.

*Castilla y la Mancha*: Francisco Javier Callejas González. Complejo hospitalario universitario de Albacete, Albacete. Angel Ortega Gonzalez. H Nuestra Señora del Prado, Talavera de la Reina, Toledo. Rosario Vargas Gonzalez, H. Virgen de la Luz, Cuenca. Encarnación López Gabaldón, Raul Hidalgo Carvajal, H. Virgen de la Salud, Toledo.

*Castilla y León*: Elena Bollo de Miguel, Silvia Fernández Huerga, Complejo Hospitalario Universitario de León. Ana Pueyo Bastida, Complejo Asistencial de Burgos, Burgos. Jesus R Hernández Hernández, Ruth Garcia García, H. Nuestra Señora de Sonsoles, Ávila. Miguel Barrueco Ferrero, Marco López Zuibizarreta, E. Consuelo Fernández, H. Universitario de Salamanca.

*Cataluña*: David De la Rosa Carrillo, H. Plató, Barcelona. Jordi Esplugas Abós, Noelia Pablos Mateos, H. Sant Joan de Déu, Martorell. Elena De Miguel Campos, H. Sant Joan de Despi, Barcelona. Pablo Rubinstein, Hospital General de Cataluña, Barcelona. Hernán Abraham Manrique Chávez, H Sagrat Cor, Barcelona. Miriam Barrecheguren, H. U.Vall d'Hebron, Barcelona.

*Valencia*: Carmen Aguar Benito, H. de Arnau de Villanova, Valencia. Pablo Catalán Serra, H. de Requena, Requena. Eusebi Chiner Vives. H. U. de San Juan, Alicante. Juan Antonio Royo Prats. H. General de Castellón, Castellón de la Plana. Cristina Sabater Abad, Esther Verdejo Mengual, H. General Universitario de Valencia. Eva Martínez- Moragon, H. Universitario Dr. Peset, Valencia.

*Extremadura*: Francisca Lourdes Marquez Perez, H. U Santa Cristina, Badajoz.

*Galicia*: Alberto Fernandez Villar, Cristina Represas Represas, Ana Priegue Carrera, Complejo hospitalario de Vigo. Marina Blanco Aparicio, Pedro Jorge Marcos Rodriguez, H. U. Juan Canalejo, La Coruña.

*Baleares*: Federico Gonzalo Fiorentino, Mª Magdalena Pan Naranjo, H. Son Espases, Palma de Mallorca. Antonia Fuster Gomila, H. Sant Llatzer, Palma de Mallorca.

*Madrid*: German Peces Barba, Felipe Villar Alvarez, Fundación Jimenez Diaz, Madrid. Carlos Jose Álvarez Martinez, H. 12 de Octubre, Madrid. Juan Luis Rodriguez Hermosa, J.L. Álvarez Sala-Walther, Elena Forcén Vicente de Vera, H. Clinico San Carlos, Madrid. José Andrés García Romero de Tejada, H. U. Infanta Sofía, San Sebastián de los Reyes, Madrid. Javier Jareño, Sergio Campos Tellez. H. Central de la Defensa, Madrid. Raul Galera Martinez, H. La Paz. Rosa Mar Gómez Punter, Emma Vázquez Espinosa, H. La Princesa, Madrid. Esther Alonso Peces, H. Principe de Asturias, Alcalá de Henares, Madrid. Juan Manuel Diez Piña, Raquel Pérez Rojo, H. U. de Móstoles, Madrid. Luis Puente Maestu, Julia Garcia de Pedro. H. U. Gregorio Marañón, Madrid. Soledad Alonso Viteri, H. U de Torrejón, Torrejón de Ardoz, Madrid.

*Navarra*: Maria Hernandez Bonaga, Complejo Hospitalario de Navarra, Pamplona.

*País Vasco*: Maria Milagros Iriberri Pascual, H de Cruces, Baracaldo. Myriam Aburto Barrenechea, H de Galdakano. Sophe Garcia Fuika, Hospital Santiago Apostol, Vitoria. Patricia Sobradillo Ecenarro, Hospital Txagorritx, Basurto.
